# Performance of MRI for standardized lymph nodes assessment in breast cancer: are we ready for Node-RADS?

**DOI:** 10.1007/s00330-024-10828-y

**Published:** 2024-06-12

**Authors:** Federica Pediconi, Roberto Maroncelli, Marcella Pasculli, Francesca Galati, Giuliana Moffa, Andrea Marra, Andrea Polistena, Veronica Rizzo

**Affiliations:** 1 Department of Radiological, Oncological, and Pathological Sciences, Sapienza—University of Rome, 00185 Rome, Italy; 2 Department of Surgery “Pietro Valdoni”, Policlinico “Umberto I”, Rome “Sapienza” University of Rome, 00128 Rome, Italy

**Keywords:** Node-RADS, Breast cancer, TNM, Lymph node invasion, Breast CE-MRI

## Abstract

**Objectives:**

The Node-RADS score was recently introduced to offer a standardized assessment of lymph node invasion (LNI). We tested its diagnostic performance in accurately predicting LNI in breast cancer (BC) patients with magnetic resonance imaging. The study also explores the consistency of the score across three readers.

**Materials and methods:**

A retrospective study was conducted on BC patients who underwent preoperative breast contrast-enhanced magnetic resonance imaging and lymph node dissection between January 2020 and January 2023. Sensitivity, specificity, positive predictive value (PPV), and negative predictive value were calculated for different Node-RADS cut-off values. Pathologic results were considered the gold standard. The overall diagnostic performance was evaluated using receiver operating characteristic curves and the area under the curve (AUC). A logistic regression analysis was performed. Cohen’s Kappa analysis was used for inter-reader agreement.

**Results:**

The final population includes 192 patients and a total of 1134 lymph nodes analyzed (372 metastatic and 762 benign). Increasing the Node-RADS cut-off values, specificity and PPV rose from 71.4% to 100% and 76.7% to 100%, respectively, for Reader 1, 69.4% to 100% and 74.6% to 100% for Reader 2, and from 64.3% to 100% and 72% to 100% for Reader 3. Node-RADS > 2 could be considered the best cut-off value due to its balanced performance. Node-RADS exhibited a similar AUC for the three readers (0.97, 0.93, and 0.93). An excellent inter-reader agreement was found (Kappa values between 0.71 and 0.83).

**Conclusions:**

The Node-RADS score demonstrated moderate-to-high overall accuracy in identifying LNI in patients with BC, suggesting that the scoring system can aid in the identification of suspicious lymph nodes and facilitate appropriate treatment decisions.

**Clinical relevance statement:**

Node-RADS > 2 can be considered the best cut-off for discriminating malignant nodes, suggesting that the scoring system can effectively help identify suspicious lymph nodes by staging the disease and providing a global standardized language for clear communication.

**Key Points:**

- *Axillary lymphadenopathies in breast cancer are crucial for determining the disease stage.*
- *Node-RADS was introduced to provide a standardized evaluation of breast cancer lymph nodes.*
- *RADS* *>* *2 can be considered the best cut-off for discriminating malignant nodes.*

## Introduction

Breast cancer (BC) is the most diagnosed cancer in the female population, with an estimated 2.3 million cases and 685,000 deaths in 2020 [1].

Tumor, lymph node, and metastasis classification (TNM) are strongly recommended before making any treatment decisions. This staging approach allows for an accurate assessment of disease extent and progression [2] and thus appropriate formulation of treatment plans, resulting in improved patient outcomes [3].

Currently, the technique used to evaluate the stage of axillary lymph nodes in BC is ultrasound, which, while appearing highly specific in the assessment of lymph nodes [4], currently has no widely used standard scores for their evaluation [5].

Traditional cross-sectional imaging modalities, such as computed tomography (CT) or contrast-enhanced-magnetic resonance imaging (CE-MRI) can also be used for lymph node staging, but these, like ultrasound, do not use standardized criteria to define the exact involvement of the nodes [3].

This lack of standardization could represent a less predictive imaging performance compared with pathology reports in BC. Nevertheless, the clinical N stage is of paramount importance for making management decisions [6].

The anatomic TNM staging system has remained unchanged from its previous versions [7]. To standardize the evaluation of lymph nodes, the eighth edition of the TNM provided more precise indications on the methods of measurement of lymph node metastases without introducing an actual diagnostic score for the evaluation of lymphadenopathy on imaging [8].

The presence of lymph node metastases is a crucial factor considered by the current cancer treatment guidelines worldwide; any macroscopic lymph-node metastasis indicates at least stage II disease [7]. Patients with locally advanced BC (stage IB-IIIC), regardless of subtype, are ideal candidates for neoadjuvant chemotherapy [9, 10]. Therefore, a precise evaluation of lymphadenopathies holds significant importance.

Elsholtz et al proposed a comprehensive scoring system, Node-RADS, based on both size and configuration criteria, to standardize the radiologic assessment of lymph node invasion (LNI) on CE-MRI scans. This scoring system is applicable to various tumor types at different anatomical sites, including regional and non-regional lymph nodes [11]. Although Node-RADS was introduced in 2021 and has shown promising results in prostate [12], bladder [13], lung [14], colon [15], and gastric [16] cancer, its role in BC has not previously been investigated. To address this gap, we conducted a retrospective review of preoperative CE-MRI performed in BC patients who underwent mastectomy or quadrantectomy with lymphadenectomy at our institution. We hypothesized that a higher Node-RADS score might be associated with an increased risk of LNI and therefore tested the overall diagnostic performance of the Node-RADS Score. The study also focused on assessing the applicability and feasibility of scoring among three readers.

## Materials and methods

### Study design and patient population

Informed consent was waived due to the retrospective nature of our study, as approved by our Institutional Review Board.

We retrospectively enrolled patients who were diagnosed with invasive carcinoma, and micro-invasive or high-risk ductal carcinoma in situ (DCIS) (G3, high ki67) [16] from January 2020 to July 2023, who underwent preoperative CE-MRI, breast surgery, and lymphadenectomy at our institution.

Those cases include patients who exhibited positive lymph nodes on imaging or were candidates for lymphadenectomy due to sentinel lymph-node failure, clinical T staging (cT) 4 tumors, or were diagnosed with inflammatory carcinoma.

**Fig. 1:**
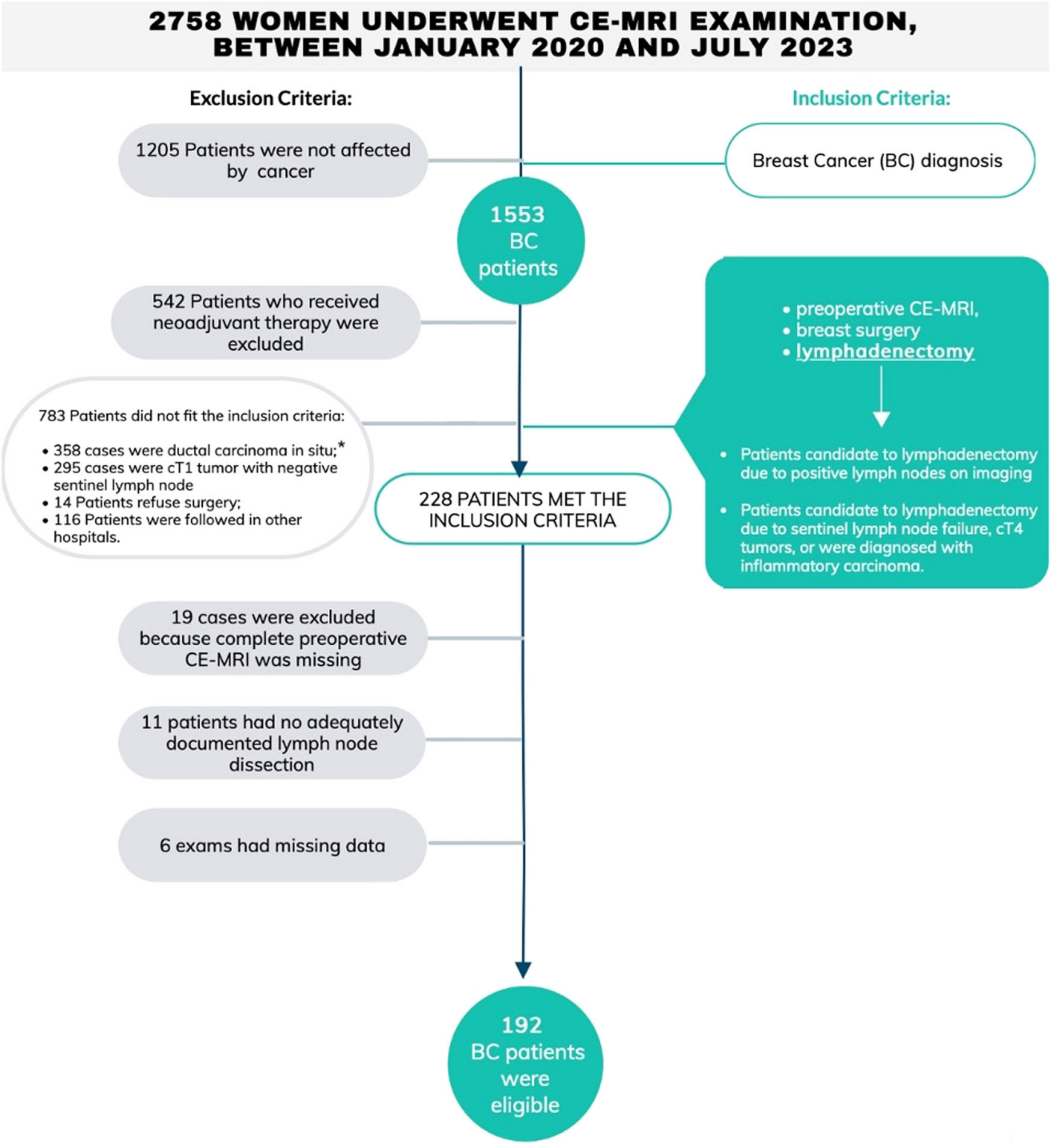
Flowchart of participants. (*) High-risk ductal carcinoma in situ (DCIS) (G3, high ki67) were included in the study

Patients who received preoperative systemic neoadjuvant therapy were excluded from the study (Fig. 1).

Furthermore, we excluded patients with incomplete preoperative CE-MRI protocol, patients without a properly documented lymph node submission, and patients with missing data.

### Baseline variables

For each patient, we extracted the following data from our prospectively maintained database: age at the time of surgery, Node-RADS suspicion degree, cT, tumor grade according to the WHO classification [17], the type of surgery performed (mastectomy or quadrantectomy), the total number of lymph nodes removed, pathologic T stage (pT), pathologic N stage (pN), and tumor classification based on the Nottingham Histologic Score [18].

### CE-MRI examination and Node-RADS assessment

CE-MRI scans were performed utilizing a 3-T magnet along with a specialized 8-channel breast coil, with patients positioned in a prone posture (Table 1).

**Table 1:** Breast CE-MRI protocol

Breast CE-MRI sequences	
Sequence	Technical characteristics
2D FSE T2-weighted FS sequence	
­RT	9000–11,000 ms
­ET	119–120 ms
­Matrix	512 × 224
­Slice thickness	3–5 mm
­FOV	350 × 350 mm
­NEX	1
­Scan time	130 s
DWI sequence	
­RT	4983–5314 ms
­ET	58 ms
­Matrix	150 × 150
­Slice thickness	3–5 mm
­FOV	350 × 350 mm
­NEX	2–2–4
­Scan time	230 s
3D GE T1-weighted FS sequences	
­Flip angle	15°
­RT	8 ms
­ET	4 ms
­Matrix	512 × 256
­Slice thickness	1.40 m
­FOV	380 × 380 mm
­NEX	1

T2-weighted sequences utilized a three-point Dixon technique (IDEAL) to achieve fat suppression. Diffusion-weighted imaging (DWI) sequences comprised *b*-values of 0, 500, and 1000 s/mm^2^, with the corresponding automatic calculation of apparent diffusion coefficient maps.

Axial dynamic 3D T1-weighted fat-suppressed sequences (DISCO) were obtained once prior to and nine times following the administration of the contrast agent, with a total acquisition duration of 120 s. Post-contrast T1-weighted images were performed after administration of 0.1 mmol/kg (0.2 mL/kg) gadolinium-based contrast agent at a rate of 3 mL/s. Gadoteridol was intravenously injected via peripheral venous access (22 gauge), followed by a 20-mL saline flush.

**Fig. 2:**
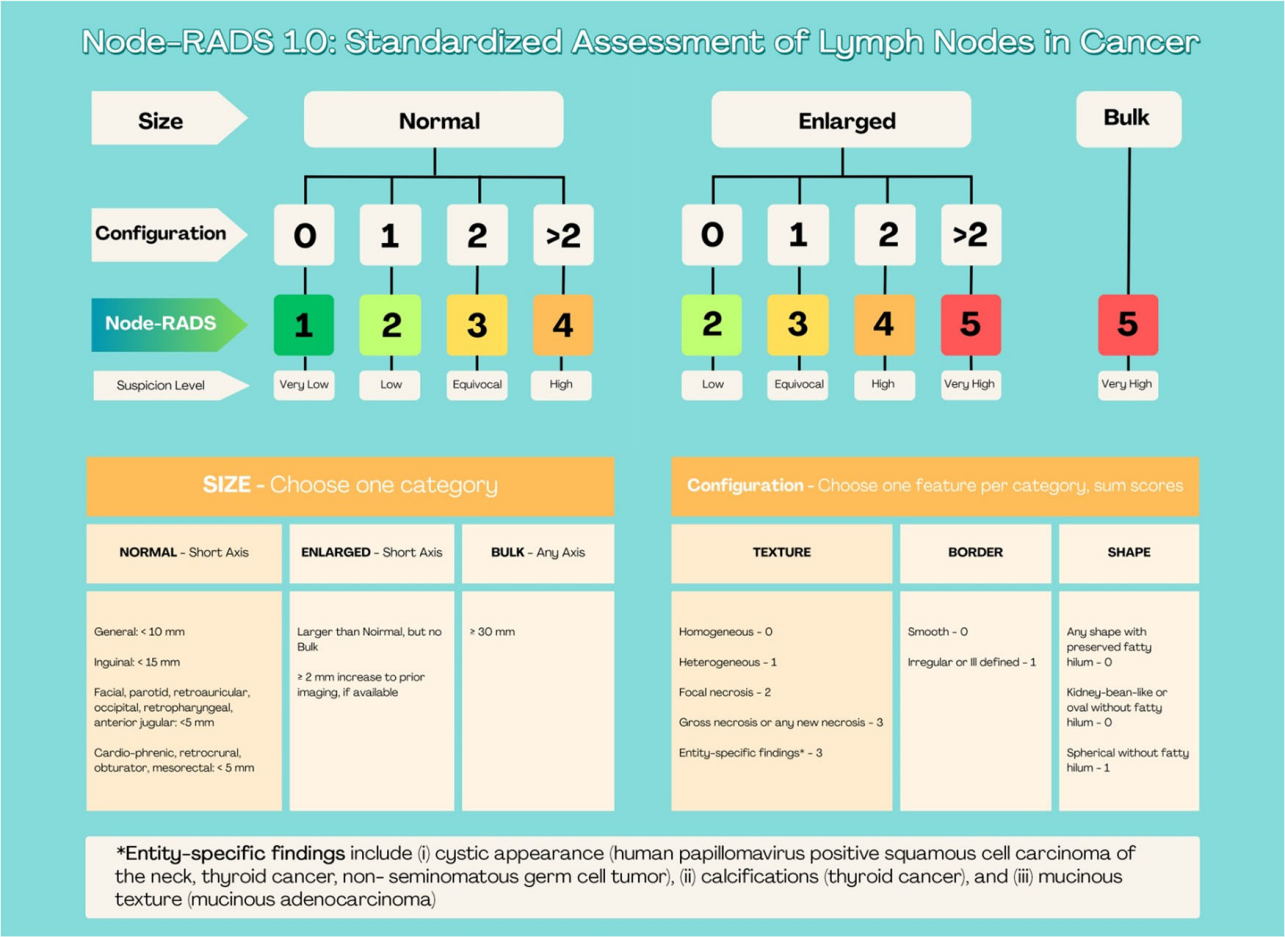
Explanation of the Node-RADS scoring system, adapted from the original publication

Subtraction images were generated for all examinations. CE-MRI images from January 2020 to July 2023 underwent retrospective evaluation. Three breast radiologists, with 15, 7, and 4 years of experience, respectively, assessed the suspicion level using Node-RADS. The images were independently reviewed by three radiologists referred to as ‘Reader 1’, ‘Reader 2’, and ‘Reader 3’. To minimize potential bias, the readers were blinded to post-operative pathological results. Image analysis adhered to Node-RADS recommendations, guided by a three-level flowchart (Fig. 2).

The evaluation of CE-MRI considered assessment categories based on size and configuration criteria. Each subcategory was assigned a score, and the cumulative score determined the likelihood of nodal involvement. The scale ranged from 1 to 5, where 1 indicated a very low likelihood and 5 indicated a very high likelihood of nodal invasion [11].

Axillary lymph nodes were classified anatomically according to the Berg classification and the Union Internationale Contra la Cancrum convention [17].

The assessment also included the supraclavicular and internal mammary nodes, which were evaluated radiologically despite not being part of the surgical procedure of axillary lymphadenectomy, as they could be involved after axillary lymph node infiltration.

Each lymph node packet was individually assessed and assigned a score based on radiological evaluation. The scores were then compared and matched with the corresponding final pathological examination results for each packet.

### Axillary lymph-node dissection and pathologic assessment

An experienced BC surgeon with at least 10 years of experience performed lymphadenectomy alongside mastectomy or quadrantectomy. The axillary dissection procedure involves ten steps, including the dissection of level-I and level-II nodes, including Rotter’s nodes. However, the standard dissection procedure does not include level-III nodes. This approach ensures the retrieval of at least ten lymph nodes, which is critical for precise staging information [19].

Level-III axillary dissection is generally performed only in cases where there is evident gross disease in level-II nodes [20].

Nodal analysis results were categorized as benign, indicative of micrometastasis (0.2–2 mm), or indicative of macrometastasis (> 2 mm). Cases of isolated tumor cells (< 0.2 mm) were classified as benign findings [21].

### Statistical analyses

For each patient, we considered the highest Node-RADS score assigned by the radiologists and then matched against the final pathology report to determine the presence or absence of lymph node involvement (pN0 vs. pN ≥ 1). To investigate trends in the rates of LNI in different categories of Node-RADS score, we performed *χ*² tests and linear by linear association.

Next, we calculated the LNI sensitivity, specificity, positive predictive value (PPV), and negative predictive value (NPV) for all possible cut-offs of the Node-RADS score (> 1 vs. > 2 vs. > 3 vs. > 4).

Specifically, the cut-off analysis was conducted by comparing the results obtained by the score 1 vs. 2, 3, 4 and 5 (cut-off > 1), the scores 1 and 2 vs. 3, 4, and 5 (cut-off > 2), the scores 1, 2, and 3 vs. 4 and 5 (cut-off > 3) and the scores 1, 2, 3, and 4 vs. 5 (cut-off > 4).

The optimal cut-off was selected by balancing sensitivity and specificity. This balance aims to ensure a reliable ability to identify suspicious lymph nodes while minimizing false positives, thus maximizing overall diagnostic accuracy.

To evaluate the diagnostic performance of the Node-RADS score for LNI, we developed receiver operating characteristic (ROC) curves and calculated the area under the curve (AUC).

Additionally, we employed univariable and multivariable logistic regression models to assess the association between the Node-RADS score and LNI. We adjusted the multivariable models for age and cT stage. The inter-reader agreement was assessed using Cohen’s kappa analysis.

The statistical significance level was set at *p* < 0.05 for all tests, and we used IBM SPSS Statistics software, version 28, for all statistical analyses and graph generation.

## Results

### Study population characteristics

From a database of 2758 women who underwent CE-MRI examination, between January 2020 and July 2023, 1553 BC patients were selected. Among these, 228 patients met the inclusion criteria.

Of these, 19 cases were excluded because complete preoperative CE-MRI was missing, 11 patients had no adequately documented lymph node dissection, and 6 exams had missing data.

Therefore, 192 female BC patients were considered eligible for this study. The median age of these patients was 56 years (30–89).

Among them, 38 patients (19.79%) underwent a mastectomy, while 154 patients (80.21%) quadrantectomy. Histological examination of surgical specimens revealed: 7 patients with high-risk DCIS, 78 patients with luminal A subtype, 39 patients with luminal B HER-, 24 patients with luminal B HER2+, 27 patients were identified as HER2−, while 17 patients were classified as triple-negative BC cases (Table 2).

**Table 2:** Demographic characteristics of enrolled patients

Demographic characteristics of enrolled patients	
Characteristic	Value (*n* = *192*)
Age (years)^a^	56 (12.77)
Sex	
Female	192 (100)
Ethnic group	
Caucasian	192 (100)
Pathologic T stage (pT)	
­Tis	7 (3.6)
­T1	71 (37)
­T2	48 (25)
­T3	53 (27.6)
­T4	13 (6.8)
Pathologic N stage (pN)	
­N0	98 (51.05)
­N1	50 (26.05)
­N2	38 (19.8)
­N3	6 (3.1)
Histologic subtypes of BC	
­DCIS	7 (3.6)
­Luminal A	78 (40.6)
­Luminal B Her 2−	39 (20.3)
­Luminal B Her 2+	24 (12.5)
­Her 2+	27 (14.1)
­Triple negative	17 (8.9)
Surgery	
­Mastectomy	38 (19.8)
­Quadrantectomy	154 (80.2)

Except where indicated, data are numbers of participants, with percentages in parentheses

^a^ Data are medians with standard deviation

### Lymph node invasion rates according to the Node-RADS Score

A total of 1134 lymph nodes were surgically removed and 372 (32.8%) were revealed to be metastatic nodes (221 macrometastatic and 151 micrometastatic) and 762 benign. The overall LNI rate was 49% (94/192), which included macrometastasis and micrometastasis.

Patients with at least one positive axillary lymph node, whether pathological supraclavicular and mammary vessel nodes associated, were considered pathological.

All the patients showed abnormal lymph nodes on the same side as the afflicted breast.

At the consensus reading of CE-MRI, we divided patients into five groups: no positive lymph nodes (31.3%), only positive axillary lymph nodes (39.6%), positive axillary and supraclavicular lymph nodes (19.8%), positive axillary and mammary vessels nodes (3.1%), and positive axillary, supraclavicular, and mammary vessels nodes (6.3%).

**Fig. 3:**
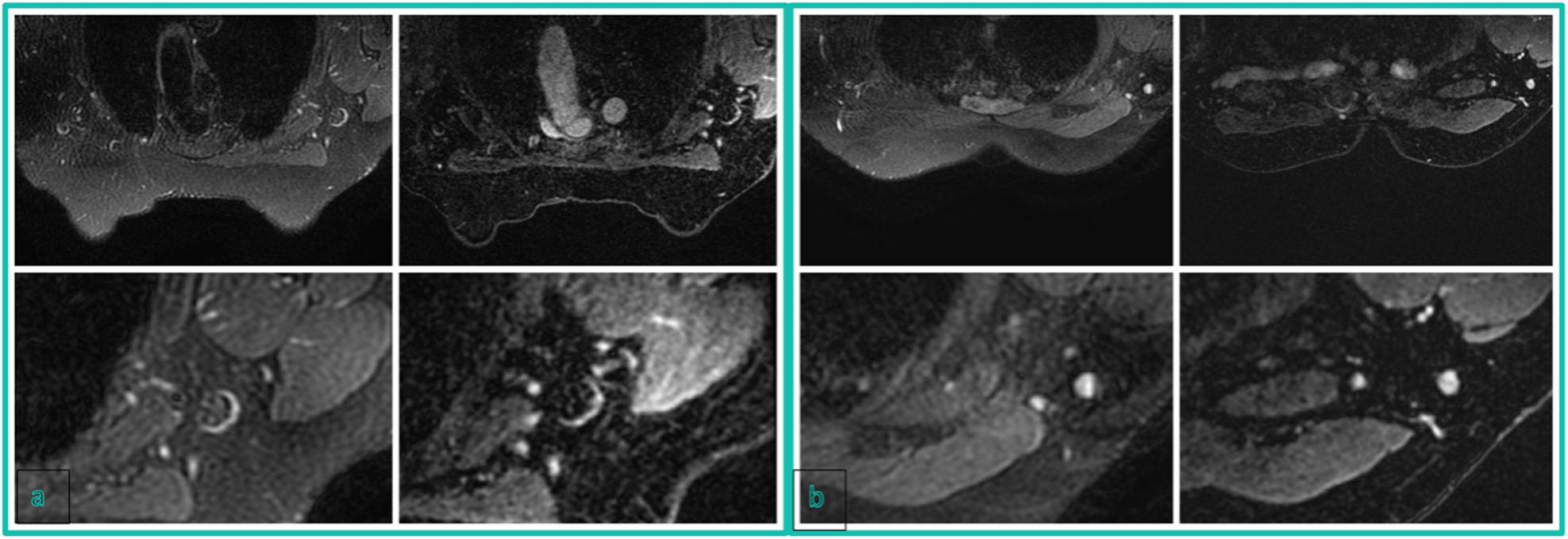
**a** Node-RADS 1 case: T2 FSE-Ideal axial sequence (on the left side) and T1 DISCO 3D axial sequence (on the right side) of a 74-year-old patient with right breast cancer and no suspicious lymph nodes. **b** Node-RADS 2 case: T2 FSE-Ideal axial sequence (on the left side) and T1 DISCO 3D axial sequence (on the right side) of a 47-years-old patient with right breast cancer and some lymph nodes characterized by normal size and spherical shape without fatty hilum

**Fig. 4:**
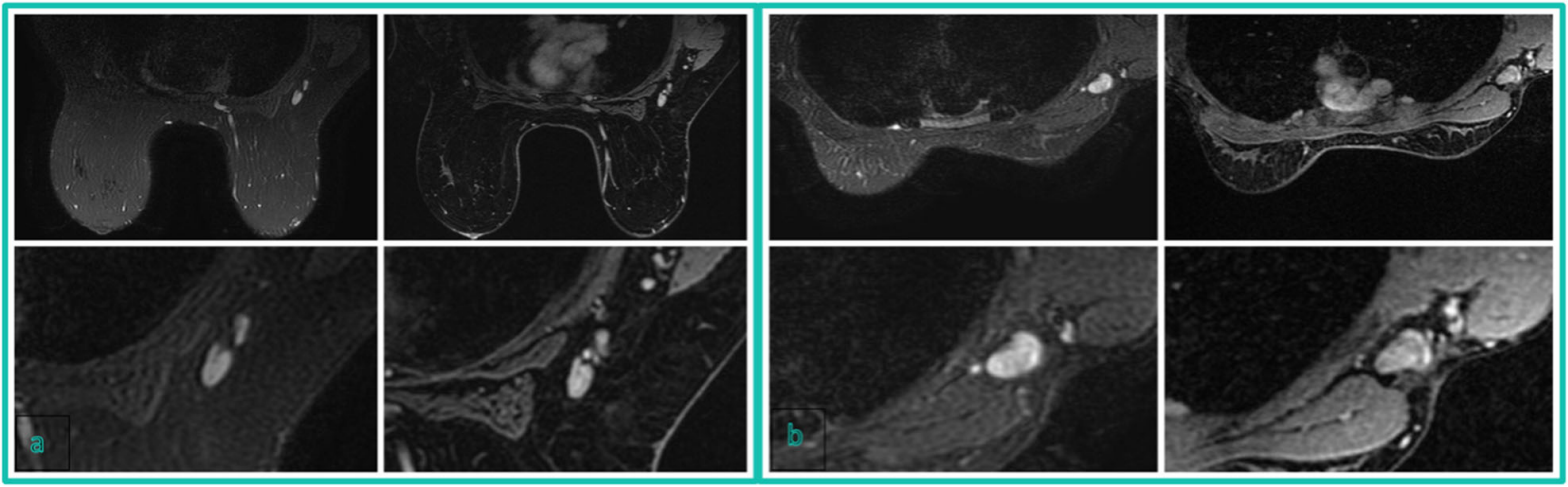
**a** Node-RADS 3 case: T2 FSE-Ideal axial sequence (on the left side) and T1 DISCO 3D axial sequence (on the right side) of a 50-years-old patient with right breast cancer and some lymph nodes characterized by enlarged size and heterogeneous texture. **b** Node-RADS 4 case: T2 FSE-Ideal axial sequence (on the left side) and T1 DISCO 3D axial sequence (on the right side) of a 43-years-old patient with right breast cancer and a lymph node characterized by enlarged size, heterogeneous texture, and spherical shape without fatty hilum

**Fig. 5:**
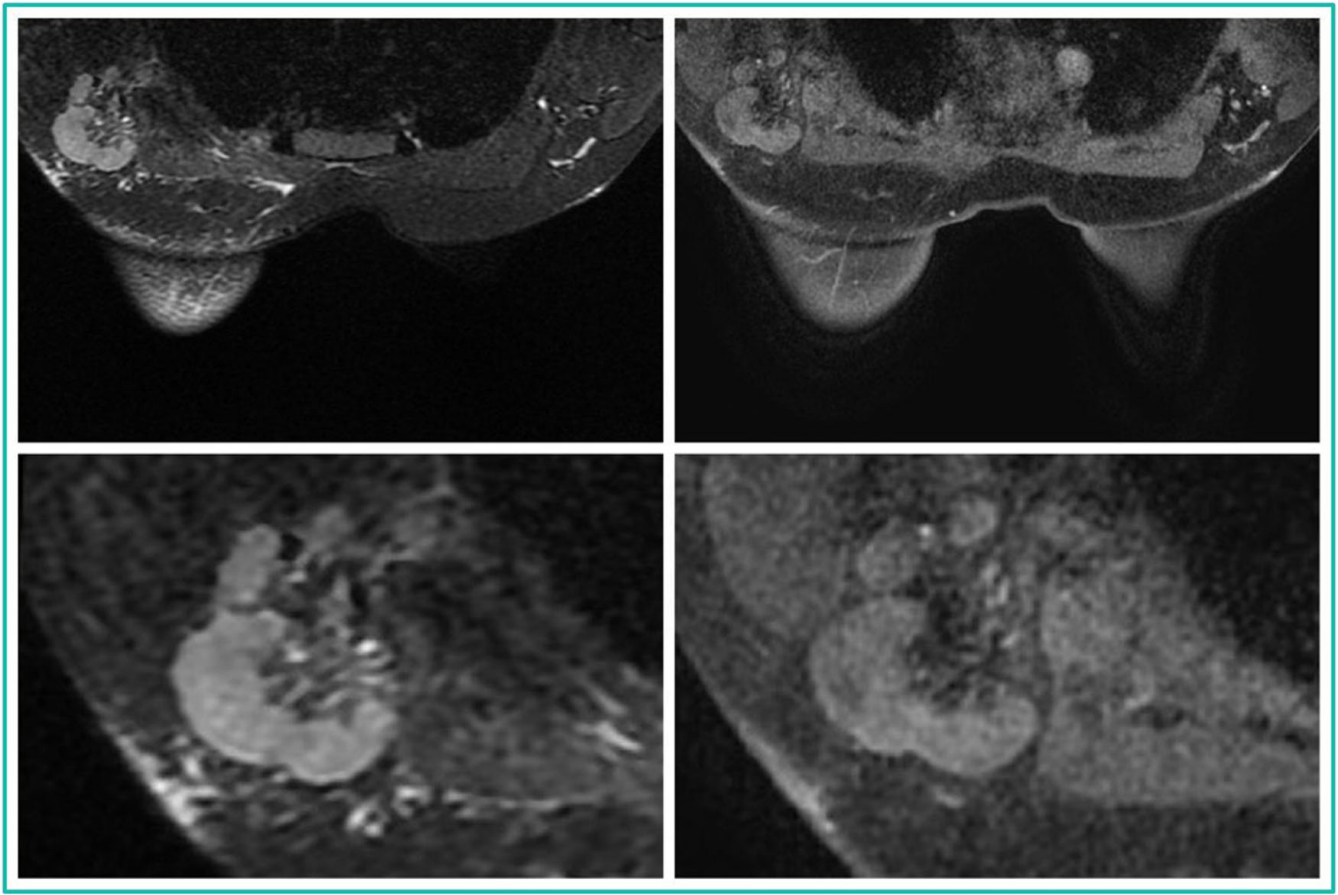
Node-RADS 5 case: T2 FSE-Ideal axial sequence (on the left side) and T1 DISCO 3D axial sequence (on the right side) of a 34-year-old patient with left breast cancer and a bulky lymph node

Based on the blinded CE-MRI image evaluation, 72 (37.5%) vs. 20 (10.4%) vs. 38 (19.8%) vs. 32 (16.7%) vs. 30 (15.6%) patients were assigned a Node-RADS score of 1 vs. 2 vs. 3 vs. 4 vs. 5, respectively, by Reader 1; 74 (38.5%) vs. 22 (10.3%) vs. 42 (21.9%) vs. 24 (12.5%) vs. 30 (15.6%) patients were assigned by Reader 2, and 67 (34.9%) vs. 26 (13.5%) vs. 32 (16.7%) vs. 33 (17.2%) vs. 34 (17.7%) patients were assigned by Reader 3 (Figs. 3–5).

The *χ*² analysis revealed a significant association between Node-RADS and LNI for all the Readers (*p* < 0.001) (Table 3).

**Table 3:** *χ*^2^ and linear by linear association

	*χ*² test	Linear by linear association
Reader 1 *p*-value	220.307 < 0.001	141.644 < 0.001
Reader 2 *p*-value	171.418 < 0.001	122.509 < 0.001
Reader 3 *p*-value	177.731 < 0.001	127.019 < 0.001

### Diagnostic performance of the Node-RADS score according to a different cut-off

**Fig. 6:**
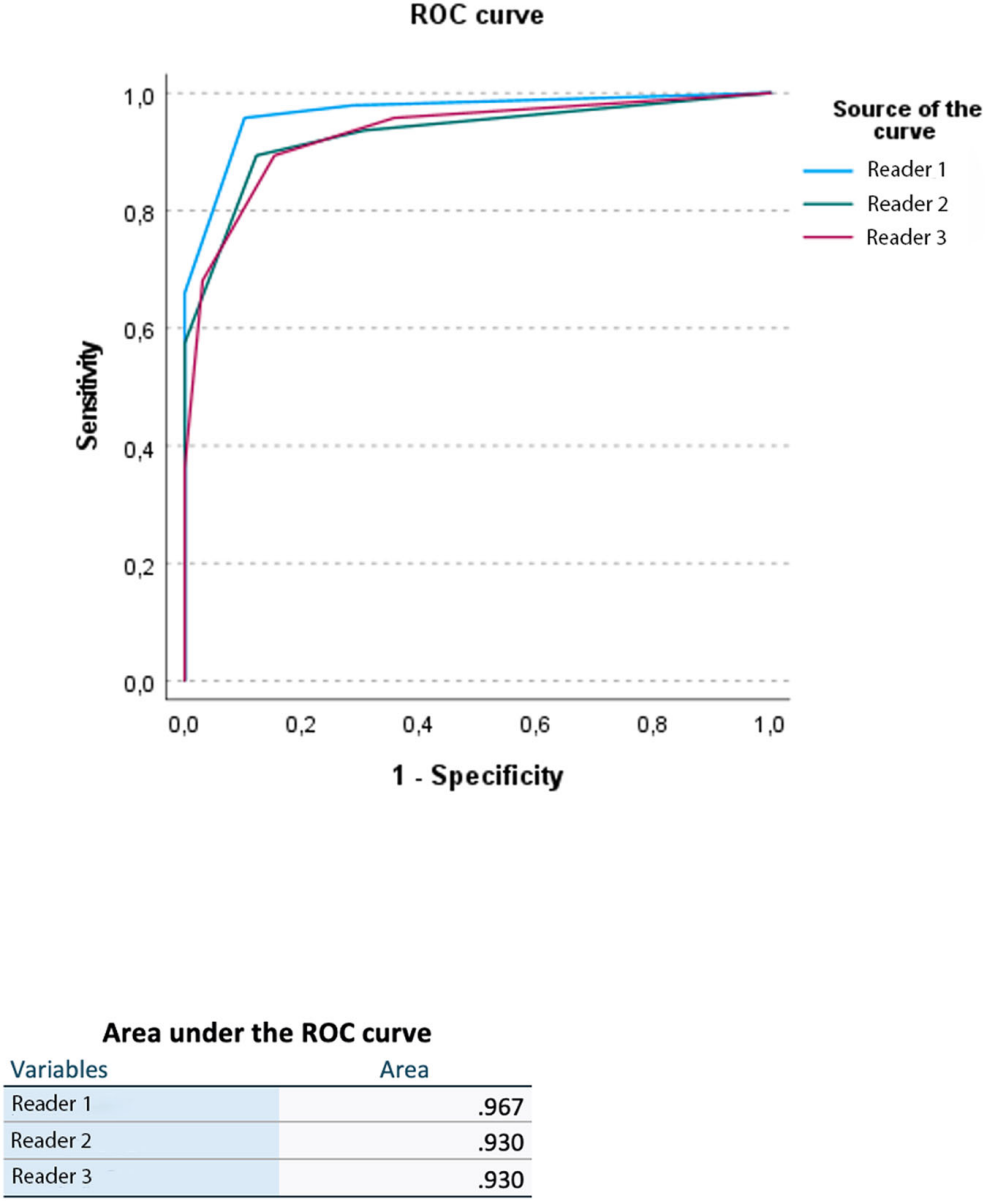
ROC curve, area under the curve (AUC), and overall model quality for Reader 1, Reader 2, and Reader 3 evaluations

Based on the ROC curve, the AUC of the Node-RADS score was 0.97 for Reader 1 and 0.93 for both Reader 2 and 3 (Fig. 6).

For Reader 1, by setting a higher Node-RADS cut-off (from 1 to 5), the specificity increased from 71.4% to 100%, and the PPV increased from 76.7% to 100%. However, the sensitivity decreased from 97.9% to 31.9%, and the NPV decreased from 97.2% to 60.5%.

For Reader 2, the specificity increased from 69.4% to 100%, and the PPV increased from 74.6% to 100% when the Node-RADS cut-off was raised from 1 to 5. Conversely, the sensitivity decreased from 93.6% to 31.9%, and the NPV decreased from 91.9% to 60.5%.

The specificity for Reader 3 went up from 64.3% to 100%, and the PPV went up from 72% to 100%. The sensitivity decreased from 95.7% to 36.2%, and the NPV decreased from 94% to 62%. Based on the balanced sensitivity and specificity values for Reader 1 (95.7% sensitivity and 89.8% specificity), Reader 2 (89.4% sensitivity and 87.8% specificity), and Reader 3 (89.4% sensitivity and 84.7% specificity). It is reasonable to conclude that a Node-RADS > 2 is the most suitable cut-off (Table 3).

### Logistic regression

In a univariable logistic regression analysis, Node-RADS correlated with LNI for Reader 1 (OR 15, 95% CI 6.77–33.23, *p* < 0.001), for Reader 2 (OR 7, 95% CI 4.2–11.5, *p* < 0.001), and for Reader 3 (OR 5.8, 95% CI 3.7–9.1, *p* < 0.001).

The variables “age” and “cT stage” were also examined in univariate logistic regression to assess their potential confounding effects. The results indicated their association with LNI (Table 3).

Following multivariable adjustments for essential confounders, Node-RADS was found to be an independent predictor of LNI (Table 4).

**Table 4:** Test accuracy and logistic regression

Test accurancy				
Reader 1				
Node-RADS	Sensitivity (CI 95%)	Specificity (CI 95%)	PPV (CI 95%)	NPV (CI 95%)
> 1	97.9 (92.4%–99.7%)	71.4 (61.4%–80.1%)	76.7 (70.6%–81.8%)	97.2 (89.8%–99.3%)
> 2	95.7 (89.5%–98.8%)	89.8 (82%–95%)	90 (83.3%–94.2%)	95.7 (89.4%–98.3%)
> 3	66 (55.5%–75.4%)	100 (96.3%–100%)	100 (94.2%–100%)	75.4 (69.8–80.2%)
> 4	31.9 (22.7%–42.3%)	100 (96.3%–100%)	100 (88.4%–100%)	60.5 (57.1%–73.3%)
**Reader 2**				
Node-RADS	Sensitivity (CI 95%)	Specificity (CI 95%)	PPV (CI 95%)	NPV (CI 95%)
> 1	93.6 (86.6%–97.6%)	69.4 (59.3%–78.3%)	74.6 (68.4%–79.9%)	91.9 (83.8%–96.1%)
> 2	89.4 (81.3%–94.8%)	87.8 (79.6%–93.5%)	87.5 (80.4%–92.3%)	89.6 (82.6%–93.9%)
> 3	57.4 (46.8%–67.6%)	100 (96.3%–100%)	100 (93.4%–100%)	71 (65.9%–75.6%)
> 4	31.9 (22.7%–42.3%)	100 (96.3%–100%)	100 (88.4%–100%)	60.5 (57.1%–63.7%)
**Reader 3**				
Node-RADS	Sensitivity (CI 95%)	Specificity (CI 95%)	PPV (CI 95%)	NPV (CI 95%)
> 1	95.7 (89.5%–98.8%)	64.3 (54%–73.7%)	72 (66.3%–77%)	94 (85.6%–97.6%)
> 2	89.4 (81.3%–94.8%)	84.7 (76%–91.2%)	84.8 (77.8%–90%)	89.2 (82.1%–93.7%)
> 3	68.1 (57.7%–77.3%)	96.9 (91.3%–99.4%)	95.5 (87.4%–98.5%)	76 (70.2%–81%)
> 4	36.2 (26.5%–46.7%)	100 (96.3%–100%)	100 (89.7%–100%)	62 (58.4%–65.5%)
**Univariate logistic regression**				
	OR (CI 95%)	*p*-value		
Age	0.966 (0.944–0.989)	0.004		
cT stage	2.495 (1.789–3.479)	< 0.001		
Node-R Reader 1	14.997 (6.769–33.228)	< 0.001		
Node-R Reader 2	6.988 (4.241–11.514)	< 0.001		
Node-R Reader 2	5.823 (3.729–9.094)	< 0.001		
**Multivariate logistic regression (enter method) Reader 1**				
	OR (CI 95%)	*p*-value		
Age	0.965 (0.919–1.014)	0.155		
cT stage	1.957 (1.005–3.813)	0.048		
Node-RADS	14.997 (6.769–33.228)	< 0.001		
**Multivariate logistic regression (enter method) Reader 2**				
	OR (CI 95%)	*p*-value		
Age	0.951 (0.913–0.990)	0.015		
cT stage	2.029 (1.005–3.813)	0.007		
Node-RADS	6.996 (4.059–12.060)	< 0.001		
**Multivariate logistic regression (enter method) Reader 3**				
	OR (CI 95%)	*p*-value		
Age	0.957 (0.919–0.996)	0.031		
cT stage	1.888 (1.133–3.146)	0.015		
Node-RADS	5.636 (3.492–9.097)	< 0.001		

*OR* odds ratio, *CI* confidence interval

### Inter-reader agreement

By comparing the readings of Node-RADS between readers, Cohen’s Kappa showed an overall agreement between Reader 1 and 2 of 0.83, between Reader 1 and 3 of 0.79, and between Reader 2 and 3 of 0.71.

The assessment of agreement among the readers within specific Node-RADS cut-off subclasses shows either good or excellent concordance (Table 5).

**Table 5:** Inter-reader agreement

Cohen’s *K* agreement					
Readers	Node-RADS	> 1	> 2	> 3	> 4
Reader 1 * Reader 2	0.834	0.934	0.917	0.803	1.000
Reader 1 * Reader 3	0.789	0.899	0.864	0.918	0.888
Reader 2 * Reader 3	0.714	0.899	0.885	0.748	0.888

## Discussion

This study showed a significant correlation between Node-RADS score and lymph node invasion. Node-RADS score > 2 was identified as the most suitable cut-off point for predicting lymph node invasion. A Node-RADS score of 3, 4, or 5 should be taken into consideration as strongly indicative of metastatic disease and this information should be considered as part of patient management.

The involvement and the number of axillary lymph nodes affected are widely recognized as crucial prognostic factors for BC. A recent meta-analysis demonstrated that the presence of occult metastases, as opposed to their absence, was significantly associated with a poorer 5-year disease-free survival (RR 1.55, 95% CI 1.32–1.82) and overall survival (RR 1.45, 95% CI 1.11–1.88) [22].

Currently, the technique used to evaluate axillary lymph-node status in BC is ultrasound. US is highly specific for the assessment of lymph nodes by using morphological criteria (round shape, absence of fatty hilum, thickening of the cortex > 3 mm) [10], raising a specificity of 98.3% in a large meta-analysis by Houssami et al [4], however, a standard score for their evaluation has not yet been created.

Cross-sectional imaging, such as CT or CE-MRI, is also important for disease evaluation and lymph node staging, but it has some limitations due to the lack of standardized criteria for defining the exact involvement of the lymph nodes [3].

Traditionally, staging has relied on the TNM staging system, which determines the “baseline risk” of BC at the time of diagnosis and after surgery [23]. Clinical nodal involvement (N stage), despite any lack of standardization, remains of paramount importance in guiding management decisions for BC patients.

Node staging plays a crucial role in predicting the likelihood of recurrence in patients who are not receiving systemic therapy. It aids in determining whether a patient should receive adjuvant chemotherapy, endocrine therapy, or anti-HER2 therapy, based on the predicted risk of recurrence [23]. This information is invaluable in tailoring personalized treatment plans for individual patients and ensuring that they receive the most appropriate and effective therapies.

The eighth edition of the TNM staging system aimed to standardize the evaluation of lymph nodes by providing more precise guidelines for the pathological measurement of lymph node metastases [8]. This included specifying different methods of approximation for the size of metastases, such as considering diameters greater than 1 mm and less than 2 mm, or equal to or greater than 2 mm. Additionally, the evaluation of lymph node clusters was addressed, emphasizing that the largest aggregate of contiguous tumor cells should be measured without including separate tumor clusters. However, the TNM staging system did not introduce a definitive diagnostic scoring system for the evaluation of lymphadenopathy based on imaging [8].

In parallel, the American College of Radiology (ACR) Breast Imaging Reporting and Data System (BIRADS) also proposed a qualitative and subjective assessment of lymph nodes. According to the ACR BIRADS guidelines, lymph nodes are described based on their size, without establishing a specific size cut-off and considering any size increase from a previous examination. Furthermore, the loss of the adipose (fatty) hilum appearance of lymph nodes and the evaluation of their margins are taken into account during the assessment [23].

These guidelines, although providing more clarity on the measurement and evaluation of lymph nodes, still rely on subjective and qualitative criteria, lacking a definitive quantitative scoring system for diagnosing lymphadenopathy through imaging.

As a result, clinical judgment and expertise continue to play a significant role in the accurate evaluation and management of lymph node involvement in BC patients, especially if ultrasound is considered the method of choice.

Recently, the Node-RADS scoring system was introduced to address this gap, providing a comprehensive evaluation of the lymph node [11].

Like other RADS, this system aims to improve the distinction between benign and malignant diseases, remove ambiguity from radiology reports, allow for automated auditing of data, and enhance clinical communication with referrers.

In previous studies focused on other parts of the body, such as the prostate, the bladder, the lung, the colon, and the stomach, the Node-RADS score has already been validated and demonstrated significant utility, with favorable outcomes [12–16] comparable to the results obtained in our current research. Moreover, the Node-RADS score emerged as an independent predictor of lymph node involvement with a moderate-to-high overall accuracy in identifying LNI. Additionally, its flexibility in allowing the establishment of different cut-off values based on specific clinical scenarios enhances its clinical applicability [12–16].

In the current study, we assessed the overall diagnostic performance of the Node-RADS scores and hypothesized that the Node-RADS score independently correlates with lymph node involvement.

The Node-RADS scores revealed a positive trend in the rates of LNI. Specifically, Node-RADS scores increased the LNI risk, establishing their status as an independent predictor even after adjusting for multiple variables (*p* < 0.001). The linear-by-linear association depicted this relationship, showing a progressive rise in LNI risk with increasing Node-RADS scores.

Based on the balanced sensitivity and specificity values for all Reader we concluded that a Node-RADS > 2 could be considered the best cut-off since, from the results obtained, it is possible to determine the presence or absence of suspicious lymph nodes.

Drawing from our collective experience, even when considering various guidelines, national consensus, and corporate recommendations, lymph nodes assigned a Node-RADS score of 1 or 2 are generally benign and do not necessitate further specific assessment.

Therefore, we recommend focusing on malignant Node-RADS scores (3, 4, and 5). For cases falling into this category, we propose a US-guided biopsy prior to surgery. This approach ensures accurate management strategies and facilitates the placement of a clip to assess the lymph nodes’ response to subsequent neoadjuvant chemotherapy.

In situations where lymph nodes receive a Node-RADS > 2 in the absence of a diagnosed breast tumor, we advise conducting a mammographic and US examination, followed by a biopsy.

The agreement among the three readers varied from good to excellent, even for the less experienced breast radiologist. The agreement values evaluated for subclasses of Node-RADS cut-offs, in most cases show even better agreement. This high level of concordance indicates that the Node-RADS scoring system can be reliably applied by different readers, enhancing its practical utility in clinical settings, including for novice radiologists.

In our study, we did not evaluate the timing for scoring, however, subjectively, in the opinion of the three readers, the average reading time per examination was the same for the Node-RADS scores and the non-standardized method of scoring nodes.

The readers found the scoring system useful in doubtful cases where the lack of standardization could lead to confusion, significantly increasing the reading times of the exam and the discrimination of suspicious lymph nodes from non-suspicious ones.

However, it is essential to acknowledge the limitations of our study, which was based on a relatively limited cohort. As such, these results warrant further validation in a larger, more diverse cohort to confirm the robustness and generalizability of the Node-RADS scoring system. Additionally, this study adopted a retrospective design, which may introduce inherent biases and limit the establishment of causal relationships between variables and outcomes.

Furthermore, the Node-RADS scoring system applicability might be specific to patients who undergo surgical treatment for BC, and caution should be exercised when extrapolating the findings to other treatment modalities or patient populations.

To the best of our knowledge, no other scoring system for lymph nodes exists and this study represents the first attempt to test the diagnostic performance of Node-RADS in surgically treated BC patients. Consequently, direct comparisons of our results with those of other studies may not be possible. Nonetheless, our findings contribute to the emerging body of knowledge regarding the role of Node-RADS in the clinical management of BC patients and underscore the need for further research to fully comprehend its potential impact on patient care.

## Conclusions

The present study serves as a fundamental step towards introducing a Node-RADS scoring system for assessing regional lymph nodes in BC patients. Notably, the Node-RADS score demonstrated its significance as an independent predictor of lymph node involvement even after adjusting for multiple variables through a multivariable analysis.

Moreover, the Node-RADS score showed moderate-to-high overall accuracy in identifying LNI, and the Node-RADS > 2 can be considered the best cut-off for discriminating malignant nodes.

This indicates that the scoring system can effectively aid in identifying suspicious lymph nodes, staging the disease, establishing a standardized language for communicating the presence of such nodes, and avoiding unnecessary biopsies. This facilitates making appropriate treatment decisions for BC patients based on the level of suspicion regarding lymph node status, suggesting no need for further evaluation for Node-RADS scores of 1 or 2, while recommending biopsy before surgery for scores of 3, 4, and 5, with the use of clip positioning.

## Abbreviations

AUC: Area under the curve
BC: Breast cancer
CE-MRI: Contrast-enhanced magnetic resonance imaging
cT: Clinical T stage
DCIS: Ductal carcinoma in situ
DWI: Diffusion-weighted imaging
LNI: Lymph node invasion
MRI: Magnetic resonance imaging
NPV: Negative predictive value
PPV: Positive predictive value
pN: Pathologic N stage
pT: Pathologic T stage
ROC: Receiver operating characteristic
TNM: Tumor, lymph node, and metastasis classification
